# Addressing epistemic injustice in HIV research: a call for reporting guidelines on meaningful community engagement

**DOI:** 10.1002/jia2.25880

**Published:** 2022-01-20

**Authors:** Marija Pantelic, Janina I. Steinert, George Ayala, Laurel Sprague, Judy Chang, Ruth Morgan Thomas, Cedric Nininahazwe, Georgina Caswell, Anders M. Bach‐Mortensen, Adam Bourne

**Affiliations:** ^1^ Brighton and Sussex Medical School University of Sussex Brighton UK; ^2^ Department of Social Policy and Intervention Oxford University Oxford UK; ^3^ Department of Social Sciences and Technology Technical University of Munich Munich Germany; ^4^ Alameda County Public Health Department San Leandro California USA; ^5^ UNAIDS Secretariat Geneva Switzerland; ^6^ International Network of People Who Use Drugs London UK; ^7^ Global Network of Sex Work Projects Edinburgh UK; ^8^ Global Network of Young People Living with HIV Cape Town South Africa; ^9^ Global Network of People Living with HIV Cape Town South Africa; ^10^ Australian Research Centre in Sex Health and Society La Trobe University Melbourne Victoria Australia; ^11^ UNSW Sydney Kirby Institute Sydney New South Wales Australia

**Keywords:** community, HIV trials, key populations, engagement, reporting guidelines, GIPA

## Abstract

**Introduction:**

Despite the widely recognized ethical and practical benefits of community engagement in HIV research, epistemic injustice persists within the field. Namely, the knowledge held by communities disproportionately affected by HIV is systematically afforded less credibility than that of more privileged academic researchers. In order to illustrate what this looks like in practice, we synthesized the extent of reporting on community engagement within recent high‐impact HIV intervention research papers. However, we also posit that the HIV research sector has the potential to devise and showcase world‐leading examples of equitable research‐community partnerships and suggest actionable key steps to achieving this goal.

**Discussion:**

In the absence of reporting requirements within the publishing process, it is difficult to infer whether and how the community have been consulted in the design, implementation, analysis and/or interpretation of findings. As an illustrative exercise, we offer a rapid synthesis of the extent of reporting on community engagement in HIV research from 2017 to 2019, which highlighted sporadic and very low rates of reporting of community engagement in recent high‐impact HIV intervention studies. Of note is that none of the included studies reported on community engagement through all stages of the research process. There were also discrepancies in how community involvement was reported. We provide three actionable recommendations to enhance reporting on community engagement in HIV research: (1) community‐led organizations, researchers and scientific journals should band together to develop, publish and require adherence to standardized guidelines for reporting on community involvement in HIV research; (2) research funders should (continue to) require details about how relevant communities have been engaged prior to the submission of funding requests; and (3) researchers should take proactive measures to describe their engagement with community organizations in a clear and transparent manner.

**Conclusions:**

There is a clear and urgent need for guidelines that facilitate transparent and consistent reporting on community engagement in HIV intervention research. Without standardized reporting requirements and accountability mechanisms within the research sector, the extent of meaningful community engagement cannot be established and may remain a catchphrase rather than reality.

## 1 INTRODUCTION

For the past four decades, people living with and affected by HIV have advocated for being meaningfully involved in the knowledge production that informs understanding of, and responses to, their health, wellbeing and survival. The HIV response has benefited from a wide range of organizations that are led by and for communities affected by HIV, including people living with HIV, sex workers, people who inject drugs, transgender people and men who have sex with men. In 1983, the Denver Principles were drafted by activists living with HIV, stating: “We condemn attempts to label us as ‘victims’, a term that implies defeat, and we are only occasionally ‘patients’, a term that implies passivity, helplessness, and dependence upon the care of others. We are ‘People With AIDS’” [1]. A decade later at the 1994 Paris AIDS Summit, heads and representatives of 42 governments agreed to “support a greater involvement of people living with HIV at all […] levels […] and to […] stimulate the creation of supportive political, legal and social environments.” This set of principles is commonly referred to as “Greater Involvement of People living with HIV/AIDS” (GIPA) and was endorsed by 189 UN member states in 2001 [2].

Today, GIPA is widely recognized as an imperative for ethical HIV research [3, 4, 5]. Principles around meaningful engagement in research have been extended to communities who are not necessarily living with but are affected by HIV [6], including sex workers, transgender people, people who use drugs, people in confined settings and men who have sex with men. These principles require that communities are meaningfully engaged throughout every phase of the research process, from defining the research questions and designing the study to analysing the data and disseminating the results. As such, meaningful engagement requires “a sustained effort which ensures that the capacity of communities involved in research is strengthened, that community members and researchers work collaboratively, and that research results benefit the community and support efforts to influence positive change” [6]. Community engagement in HIV research also ensures that findings are relevant to the end users of the resulting testing, prevention and treatment delivery mechanisms. A growing body of evidence suggests that community engagement in research is associated with improved study outcomes, intervention uptake, applicability of findings to real‐world implementation and improved dissemination of findings [7].

This commentary argues that despite the widely recognized ethical and practical benefits of community engagement in HIV research, epistemic injustice persists within the field. Epistemology is the science of knowledge, and epistemic injustice occurs when the knowledge held by communities disproportionately affected by HIV is “systematically afforded less credibility” than that of more privileged academic researchers [8]. Examples of this include research where community input is solicited but not acknowledged, where community input is only partially solicited without meaningful involvement in all stages of the research process; or where input is not solicited at all. As such, epistemic injustice can perpetuate the marginalization of communities affected by HIV and lead to poorer quality research.

To illustrate what this looks like in practice, we synthesized the extent of reporting on community engagement within recent high‐impact HIV intervention research papers. We suggest actionable steps to achieving equitable research‐community partnerships in the HIV sector.

### 1.1 Co‐production of this commentary

This commentary was conceptualized and written in a collaborative effort between community experts, activists and academic researchers. The team consisted of experts from the Global Network of People Living with HIV (GNP+), the Global Network of Young People Living with HIV (Y+), the International Network of People Who Use Drugs (INPUD), the Global Network of Sex Work Projects (NSWP), MPact Global Action for Gay Men's Health and Rights (formerly the Global Forum on MSM and HIV) and UNAIDS, as well as HIV researchers working on community engagement in the HIV response.

## 2 DISCUSSION

As a basic premise, we contend that all research working on issues relating to HIV among key populations should engage with affected communities and their representatives at all stages of the research process. While researchers are expected to meaningfully engage with communities affected by HIV, there are currently no reporting requirements within the publishing process. The lack of accountability on community engagement makes it difficult to infer whether and how the community have been consulted in the design, implementation, analysis and/or interpretation of findings.

We make no claim that those authors who do not report on community engagement did not do so but instead seek to emphasize the lack of transparency on this, as a first step to overcoming epistemic injustice.

The last decade of the HIV pandemic has seen the development of guidelines, resources and action plans by national and parastatal organizations that aim to facilitate community engagement in HIV‐related research, especially among those populations most affected. While varying in tone and detail, all emphasize the need for research to inform HIV prevention, testing and treatment interventions so as to ensure they sufficiently address the issues of critical importance in an ethical and pragmatic manner. As outlined in the 2011 UNAIDS guidelines on good participatory practice for biomedical HIV prevention trials, such engagement should span the entirety of the research process, from formative research activities through to protocol development, data collection, analysis, publication and dissemination [9]. The same document emphasizes how meaningful community engagement can help ensure that research questions and procedures are culturally sensitive and appropriate, thus improving recruitment, retention, adherence and other trial outcomes. More recent guidelines on community engagement with gay men and other men who have sex with men also acknowledge how meaningful engagement with those most affected can improve the quality of research, its uptake and implementation [6]. This may be particularly salient in contexts where key populations are criminalized and where strong, clear advocacy is required to help effect change.

### 2.1 Epistemic injustice: extent of reporting on community engagement in high‐impact HIV research

Without standardized reporting requirements and accountability mechanisms within the research sector, meaningful community engagement is likely to remain a catchphrase rather than reality. In order to illustrate the need for greater transparency on the level of community engagement in HIV intervention research, we reviewed studies published between 2017 and 2019 that evaluated the effectiveness of interventions aiming to improve uptake, use and/or adherence to efficacious HIV prevention, testing and treatment tools among key populations. We chose this period because of the high number of trials conducted in various parts of the world to determine what works to improve uptake of and adherence to antiretroviral medications for both prevention and treatment [10, 11]. These trials informed World Health Organization guidelines, further influencing HIV‐related policy and practice to date [12, 13, 14].

We included studies evaluating the effectiveness of interventions aiming to improve uptake, use and/or adherence to efficacious technologies for HIV prevention, testing and treatment among people living with HIV and key populations, from four highest impact factor journals in the fields of medicine and HIV (*n* = 8). Efficacious tools included: condoms, sterile injecting equipment, pre‐exposure prophylaxis, post‐exposure prophylaxis, HIV testing, any anti‐retroviral treatment regimens and prevention of vertical HIV transmission. Studies on basic science (i.e. virology) were excluded. We only included quantitative longitudinal study designs due to their capacity to determine the effectiveness of interventions.

**Figure 1:**
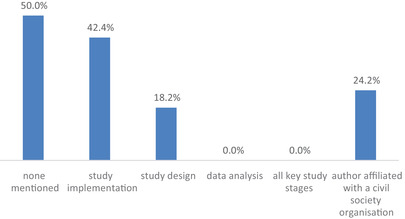
Extent of reporting on community engagement in HIV research (*n* = 66 publications).

After screening 940 articles, 66 publications from Australia, Botswana, Brazil, China, Congo, El Salvador, Estonia, Guatemala, India, Kenya, Lesotho, Nigeria, Norway, Peru, South Africa, Tanzania, Uganda, the United States, Zambia and Zimbabwe were included (please see the Table S1 for the full article list). None of the included studies reported community involvement in all key stages of the research process, from conceptualization to data analysis (Figure 1). Thirty‐three studies (50.0%) did not mention any community involvement at all. Of the 33 studies that reported on community involvement in the research process, we found notable inconsistency in the extent and manner in which this was described.

Of the reviewed papers, 12 (18.2%) reported some community involvement in the design of the study (Figure 1), mainly: intervention design (all 12 studies); the development of questionnaires used for evaluating the intervention [15]; and selection of study sites [16]. Despite few studies reporting on community involvement in the design of the research, we found that 28 included papers (42.4%) reported on community involvement during study implementation (Figure 1), engaging community members as data collectors, peer counsellors, providers of space, participant recruiters or peer educators. None of the 66 studies reported whether or how communities were involved in the data analysis or interpretation of findings (Figure 1).

This reporting stands in contrast to aforementioned guidance on community engagement, which encourages consultation from the outset to ensure the research meets salient need [6]. It also highlights the need for items on community engagement to be included in standardized reporting guidelines for HIV intervention research. None of the reviewed papers reported on the terms under which community representatives or peers were employed, for example how much they were paid or the length of their contract. However, based on what was reported, communities are likely to be engaged for less senior, more time‐consuming and lower‐paid jobs (such as data collection and peer outreach).

Sixteen of the 66 reviewed papers (24.2%) included authors with affiliations to civil society organizations (Figure 1). This suggests that there may have been some community involvement in the paper write‐up and interpretation of findings. Within their acknowledgement sections, 16 papers thanked community organizations for their involvement in the studies. Of these, seven studies did not report on community engagement in the design or implementation of the study. There are a number of scenarios that could explain this discrepancy: (1) that community engagement in HIV intervention research conducted between 2017 and 2019 may have occurred more frequently than was reported; (2) that communities were being acknowledged for their otherwise unmentioned labour; or (3) that acknowledging community organizations may have been perceived as a politically correct thing to do, even in the absence of meaningful involvement. Alternatively, the organizations credited may not have been involved as partners but rather as subjects of the research or sources for recruiting participants. Additionally, it was unclear whether the listed community organizations were professionalized non‐governmental organizations serving communities or community‐led organizations. These findings further underscore the need for reporting standards on community engagement in research processes.

### 2.2 Call to action: key tasks for researchers, scientific journals and research funders

There is an urgent need for clearly articulated guidelines that could facilitate transparent and consistent reporting on community engagement in HIV intervention research. Here, we provide three actionable recommendations to enhance reporting on community engagement in HIV research:

1. *Community‐led organizations, researchers and scientific journals should collectively develop, publish and require adherence to standardized guidelines for reporting on community involvement in HIV research*. This recommendation is in line with previous calls for metrics to help track community engagement in global health research [17, 18]. There is precedent for this; in 2017, Staniszewska and colleagues published the first international Guidance for Reporting Involvement of Patients and the Public (GRIPP2) [19], which prompted the British Medical Journal (BMJ) to “request that [submitting] authors provide a Patient and Public Involvement statement in the methods section of their papers” [20]. However, standardized reporting guidelines for trials, which are meant to improve the quality and transparency of the research process, currently do not include any requirements to explain whether and how communities were involved in the study [21].

Future enhanced guidelines for quantitative research reporting should draw on existing best practices, including those from qualitative research, which has traditionally emphasized community engagement to a greater extent. To remove ambiguity, reporting guidelines should include explicit statements pertaining to the level of community involvement in study conceptualization, recruitment (such as making explicit efforts to attract and empower people living with and at high risk for HIV in hiring processes), analysis and write‐up. To accommodate the need for more detailed reporting, journals may consider increasing word count allowances and integrating reporting requirements for community involvement in their author guidelines.

2. *Given that conceptualization of research occurs at or before the grant writing stage, research funders should (continue to) require details about how relevant communities have been engaged prior to the submission of funding requests and how their participation in the proposed research will be resourced*. We found few intervention studies reported on community engagement in the conceptualization stage of studies. Community engagement in research is not cost neutral and few community organizations are core funded to engage with researchers. Research funders can address this by actively making provisions for meaningful community involvement in their funding structures, including as grants or consultancies to community organizations to ensure their involvement. Research funders could also require open science practices during the grant writing process itself, which would encourage open exchanges of research ideas between scholars and civil society organizations at an early stage.

3. *In the absence of reporting standards or accountability mechanisms among the funders and publishers of research, researchers should take proactive measures to describe their engagement with community organizations in a clear and transparent manner* – within conceptualization, design, delivery, analysis and interpretation phases, in line with existing guidance [5, 6, 22, 23, 24]. Such an approach helps to ensure the value of the research and further facilitates the translation of research findings into advocacy and action. Based on what is currently being reported in research papers, community involvement in research is likely to be occurring within power‐imbalanced scenarios, which is not unique to the HIV sector [8]. This could be overcome in the medium‐ and longer‐term through engaging community partners as co‐principal investigators or co‐investigators from the outset; naming community‐based organizations as partners in grant proposals, with clarity on whether these organizations are community‐led; agreeing to principles of engagement that are project specific and are negotiated ahead of time; setting up memorandums of understanding; building capacity strengthening plans that are bidirectional; and building equitable budgets and plans for division of labour.

## 3 CONCLUSIONS

We make no claim that the authors of the 66 papers reviewed as part of this commentary did not engage communities in their research but rather note the absence of clear and consistent reporting as to whether and how this is occurring. The lack of reporting guidelines on community engagement HIV intervention research brings to question the validity, suitability and relevance of the very interventions that are considered cutting edge and evidence based by the HIV scientific community. Without enhanced accountability mechanisms, epistemic injustice persists despite substantial progress towards community engagement in the HIV response. This is because the lack of reporting on community engagement often makes it impossible to infer whether and how the community have been consulted in the design, implementation, analysis and/or interpretation of findings, further fuelling power imbalances in the knowledge production process.

Many academic researchers, including those co‐authoring this commentary, have failed to report on community engagement when it did occur due to a lack of accountability mechanisms and requirements to report on other aspects of the study methodology within tight word limits. Reporting guidelines and accountability mechanisms enforced by journals have the potential to make us all do better, which we must.

## COMPETING INTERESTS

The authors have no competing of interests.

## AUTHORS’ CONTRIBUTIONS

All authors contributed to the conceptualization of the paper, interpretation of findings and resultant recommendations, as well as the manuscript write up. MP led on the paper and analysed the data with feedback from all authors. JIS and AMBM ran the searches, screened articles and extracted the data, with contributions from MP. All authors have read and approved the final manuscript.

## AUTHOR INFORMATION

MP is a Lecturer in Public Health at the Brighton and Sussex Medical School, University of Sussex; and Associate Member at the Department of Social Policy and Intervention, University of Oxford. GA is the Deputy Director of the Alameda County Public Health Department and former Executive Director (ED) of MPact Global Action for Gay Men's Health and Rights. LS is the Chief of the UNAIDS Community Mobilization, Community Support, Social Justice and Inclusion Department, and was ED of the Global Network of People Living with HIV (GNP+) during the conceptualization of this review. JC is the ED of the International Network of People Who Use Drugs. RMT is the Global Coordinator of the Network of Sex Work Projects. GC is Head of Programmes at GNP+. CN is the Programmes Manager at the Global Network of Young People Living with HIV. JIS is a Lecturer in Global Health at the TUM School of Governance, Technical University of Munich, and Associate Member at the Department of Social Policy and Intervention, University of Oxford. AMBM is a Carlsberg Foundation visiting research fellow at the Department of Social Policy and Intervention, University of Oxford. AB is Associate Professor of Public Health and Deputy Director of the Australian Research Centre in Sex, Health and Society at La Trobe University, Melbourne as well as Senior Visiting Fellow at the Kirby Institute, UNSW Sydney.

## FUNDING

Funding for preliminary searches and article screening was provided by Frontline AIDS. No other parts of this review were funded.

## DISCLAIMER

The views expressed are solely those of the authors and do not represent those of UNAIDS.

## Supporting information


**Table S1**. Studies included in the rapid review

## Data Availability

This commentary did not involve collection or analysis of primary data. Data extracted from primary studies as part of the rapid review are available upon request.
