# Supplementary material for: Addressing epistemic injustice in HIV research: a call for reporting guidelines on meaningful community engagement
Source: J Int AIDS Soc. 2022 Jan 20;25(1):e25880. doi: 10.1002/jia2.25880 (PMC8771147; doi:10.1002/jia2.25880)
Supplement: Supplementary file 1 — Table S1. Studies included in the rapid review [file JIA2-25-e25880-s001.docx]

**Supplementary Material**

Supplemental table: Studies included in the rapid review

| Title and year | First author | Country |
| --- | --- | --- |
| Venue‐Based HIV‐Testing: An Effective Screening Strategy for High‐Risk Populations in Lima, Peru (2018) | Allan‐Blitz et al. | Peru |
| Acceptability and Preliminary Efficacy of an Online HIV Prevention Intervention for Single Young Men Who Have Sex with Men Seeking Partners Online: The myDEx Project (2019) | Bauermeister et al. | USA |
| Who Do You Reach? A Norwegian Pilot Project on HIV Self‐Testing that Targeted Men Who Have Sex with Men (2019) | Bjørnshagen et al. | Norway |
| Project nGage: Results of a Randomized Controlled Trial of a Dyadic Network Support Intervention to Retain Young Black Men Who Have Sex With Men in HIV Care (2017) | Bouris et al. | USA |
| Peer support and the HIV continuum of care: results from a multi-site randomized clinical trial in three urban clinics in the United States (2018) | Cabral et al. | USA |
| "The Metropolitan Atlanta community adolescent rapid testing initiative study: closing the gaps in HIV care among youth in Atlanta, Georgia, USA" (2017) | Camacho-Gonzales et al. | USA |
| Recalling, Sharing and Participating in a Social Media Intervention Promoting HIV Testing: A Longitudinal Analysis of HIV Testing Among MSM in China (2019) | Cao et al. | China |
| A Randomized Controlled Pilot Study of a Culturally-Tailored Counseling Intervention to Increase Uptake of HIV Pre-exposure Prophylaxis Among Young Black Men Who Have Sex with Men in Washington, DC (2019) | Desrosiers et al. | USA |
| Intervention Reach and Sexual Risk Reduction of a Multi‐level, Community‐Based HIV Prevention Intervention for Crack Users in San Salvador, El Salvador (2018) | Dickson‐Gomez et al. | El Salvador |
| Past Care Predicts Future Care in Out‐of‐Care People Living with HIV: Results of a Clinic‐Based Retention‐in‐Care Intervention in North Carolina (2018) | Donovan et al. | North Carolina |
| The Influence of Internalized Stigma on the Efficacy of an HIV Prevention and Relationship Education Program for Young Male Couples (2018) | Feinstein et al. | Chicago |
| Does Economic Strengthening Improve Viral Suppression Among Adolescents Living with HIV? Results From a Cluster Randomized Trial in Uganda (2018) | Gauer Bermudez et al. | Uganda |
| Addressing Early Retention in Antenatal Care Among HIV- Positive Women Through a Simple Intervention in Kinshasa, DRC: The Elombe ‘‘Champion’’ Standard Operating Procedure (2017) | Gill et al. | Kinshasa, Congo |
| A Randomized Controlled Trial of Intensive Case Management (Project Bridge) for HIV‐Infected Probationers and Parolees (2017) | Gordon et al. | USA |
| Alignment of adherence and risk for HIV acquisition in a demonstration project of pre-exposure prophylaxis among HIV serodiscordant couples in Kenya and Uganda: a prospective analysis of prevention-effective adherence (2017) | Haberer et al. | Kenya |
| Enhanced Prophylaxis plus Antiretroviral Therapy for Advanced HIV Infection in Africa (2017) | Hakim et al. | Uganda, Zimbabwe, Malawi, and Kenya |
| "Implementation of a comprehensive safer conception intervention for HIV-serodiscordant couples in Kenya: uptake, use and effectiveness" (2019) | Heffron et al. | Kenya |
| Achieving the first 90 for key populations in sub-Saharan Africa through venue-based outreach: challenges and opportunities for HIV prevention based on PLACE study findings from Malawi and Angola (2018) | Herce et al. | Urban Malawi and Angola |
| High pre-exposure prophylaxis uptake and early adherence among men who have sex with men and transgender women at risk for HIV Infection: the PrEP Brasil demonstration project (2017) | Hoagland et al. | Brasil |
| Project ACCEPT: Evaluation of a Group‑Based Intervention to Improve Engagement in Care for Youth Newly Diagnosed with HIV (2018) | Hosek et al. | USA |
| Targeted HIV testing at birth supported by low and predictable mother-to-child transmission risk in Botswana (2018) | Ibrahim et al. | Botswana |
| A Randomized Controlled Trial to Increase HIV Testing Demand Among Female Sex Workers in Kenya Through Announcing the Availability of HIV Self-testing Via Text Message (2019) | Kelvin et al. | Kenya |
| A Randomized Controlled Trial to Increase HIV Testing Demand Among Female Sex Workers in Kenya Through Announcing the Availability of HIV Self‐testing Via Text Message (2019) | Kelvin et al. | Kenya |
| A Strengths-Based Case Management Intervention to Reduce HIV Viral Load Among People Who Use Drugs (2018) | Kral et al. | USA |
| Use of an mHealth Intervention to Improve Engagement in HIV Community‐Based Care Among Persons Recently Released from a Correctional Facility in Washington, DC: A Pilot Study (2019) | Kuo et al. | USA |
| Effect of Offering Same-Day ART vs Usual Health Facility Referral During Home-Based HIV Testing on Linkage to Care and Viral Suppression Among Adults With HIV in Lesotho: The CASCADE Randomized Clinical Trial (2018) | Labhardt et al. | Lesotho |
| Mechanism of Change in Cognitive Behavioral Therapy for Body Image and Self‑Care on ART Adherence Among Sexual Minority Men Living with HIV (2018) | Lamb et al. | USA |
| Evaluation of Project RISE, an HIV Prevention Intervention for Black Bisexual Men Using an Ecosystems Approach (2018) | Lauby et al. | USA |
| Peer counselling versus standard-of-care on reducing high-risk behaviours among newly diagnosed HIV-positive men who have sex with men in Beijing, China: a randomized intervention study (2018) | Liu et al. | China |
| Making the Link: A Pilot Health Navigation Intervention to Improve Timely Linkage to Care for Men Who have Sex with Men and Transgender Women Recently Diagnosed with HIV in Guatemala City (2019) | Loya‐Montiel et al. | Guatemala |
| Emtonjeni-A Structural Intervention to Integrate Sexual and Reproductive Health into Public Sector HIV Care in Cape Town, South Africa: Results of a Phase II Study (2017) | Mantell et al. | South Africa |
| Cash versus food assistance to improve adherence to antiretroviral therapy among HIV-infected adults in Tanzania: a randomized trial (2018) | McCoy et al. | Tanzania |
| Community-based accompaniment with supervised antiretrovirals for HIV-positive adults in Peru: a cluster-randomized (2018) | McLaughlin et al. | Peru |
| Comparison of Home-Based Oral Fluid Rapid HIV Self-Testing Versus Mail-in Blood Sample Collection or Medical/Community HIV Testing By Young Adult Black, Hispanic, and White MSM: Results from a Randomized Trial (2019) | Merchant et al. 2019 | USA |
| A scalable, integrated intervention to engage people who inject drugs in HIV care and medication-assisted treatment (HPTN 074): a randomised, controlled phase 3 feasibility and efficacy study (2018) | Miller et al. | Ukraine, Vietnam and Indonesia |
| A Pilot Randomized Controlled Trial of an Integrated In-person and Mobile Phone Delivered Counseling and Text Messaging Intervention to Reduce HIV Transmission Risk among Male Sex Workers in Chennai, India (2017) | Mimiaga et al. | India |
| Pilot randomisex controlled trial of acceptance-based behaviour therapy to promote HIV acceptance, HIV disclosure and retention in medical care (2017) | Moitra et al. | USA |
| A Network Intervention to Locate Newly HIV Infected Persons Within MSM Networks in Chicago (2019) | Morgan et al. | USA |
| Changes in engagement in HIV prevention and care services among female sex workers during intensified community mobilization in 3 sites in Zimbabwe, 2011 to 2015 (2018) | Ndori-Mharadze et al. | Zimbabwe |
| Randomized controlled trial of a community-based intervention on HIV and nutritional outcomes at 6 months among women living with HIV/AIDS in rural India (2018) | Nyamathia et al. | India |
| Behavioral Changes Following Uptake of HIV Pre-exposure Prophylaxis Among Men Who Have Sex with Men in a Clinical Setting (2018) | Oldenburg et al. | USA |
| Effect of HIV self-testing on the number of sexual partners among female sex workers in Zambia (2018) | Oldenburg et al. | Zambia |
| Couples-Focused Prevention Program to Reduce HIV Risk Among Transgender Women and Their Primary Male Partners: Feasibility and Promise of the Couples HIV Intervention Program (2018) | Operario et al. | USA |
| The Effect of HIV Self-Testing Delivery Models on Female Sex Workers’ Sexual Behaviors: A Randomized Controlled Trial in Urban Uganda (2019) | Ortblad et al. | Uganda |
| Testing the Efficacy of Combined Motivational Interviewing and Cognitive Behavioral Skills Training to Reduce Methamphetamine Use and Improve HIV Medication Adherence Among HIV‐Positive Gay and Bisexual Men (2018) | Parsons et al. | USA |
| Optimizing linkage to care and retention on treatment of adolescents with newly diagnosed HIV infection (2017) | Ruira et al. | Kenya |
| Brief counselling after home-based HIV counselling and testing strongly increases linkage to care: a cluster-randomized trial in Uganda (2017) | Ruzagira et al. | Uganda |
| The Impact of Cell Phone Support on Psychosocial Outcomes for Youth Living with HIV Nonadherent to Antiretroviral Therapy (2018) | Sayegh et al. | USA |
| Enhancing Adherence to Care in the HIV Care Continuum: The Barrier Elimination and Care Navigation (BEACON) Project Evaluation (2018) | Shacham et al. | USA |
| A Pilot Study of “Peer Navigators” to Promote Uptake of HIV Testing, Care and Treatment Among Street-Connected Children and Youth in Eldoret, Kenya (2019) | Shah et al. | Kenya |
| Community intervention improves knowledge of HIV status of adolescents in Zambia: findings from HPTN 071-PopART for youth study (2017) | Shanaube et al. | Zambia |
| Integrated Bio-behavioral Approach to Improve Adherence to Pre-exposure Prophylaxis and Reduce HIV Risk in People Who Use Drugs: A Pilot Feasibility Study (2018) | Shrestha et al. | USA |
| Improving AIDS Care After Trauma (ImpACT): Pilot Outcomes of a Coping intervention Among HIV‑Infected Women with Sexual Trauma in South Africa (2018) | Sikkema et al. | South Africa |
| Drug Use and HIV Prevention With Young Gay and Bisexual Men: Partnered Status Predicts Intervention Response (2018) | Starks & Parsons | USA |
| A Pilot Randomized Trial of Intervention Components Addressing Drug Use in Couples HIV Testing and Counseling (CHTC) with Male Couples (2019) | Starks et al. | USA |
| A Randomized Controlled Trial of a Rapid Re‐housing Intervention for Homeless Persons Living with HIV/AIDS: Impact on Housing and HIV Medical Outcomes (2019) | Towe et al. | USA |
| Uptake of HIV self-testing and linkage to treatment among men who have sex with men (MSM) in Nigeria: A pilot programme using key opinion leaders to reach MSM (2018) | Tun et al. | Nigeria |
| Effects of Counselling on Adherence to Antiretroviral Treatment Among People with HIV in Estonia: A Randomized Controlled Trial (2018) | Uusküla et al. | Estonia |
| Predictors of Daily Adherence to HIV Pre‐exposure Prophylaxis in Gay/ Bisexual Men in the PRELUDE Demonstration Project (2018) | Vaccher et al. | Australia |
| Continuous quality improvement intervention for adolescent and young adult HIV testing services in Kenya improves HIV knowledge (2017) | Wagner et al. | Kenya |
| Effects of Depression Alleviation on ART Adherence and HIV Clinic Attendance in Uganda, and the Mediating Roles of Self-Efficacy and Motivation (2017) | Wagner et al. | Uganda |
| "A Randomized Controlled Trial Evaluating Efficacy of Promoting a Home-Based HIV Self-Testing with Online Counseling on Increasing HIV Testing Among Men Who Have Sex with Men" (2017) | Wang et al. | Hong Kong |
| Distribution of HIV Self-tests by HIV-Positive Men Who Have Sex with Men to Social and Sexual Contacts (2019) | Wesolowski et al. | USA |
| Effect of Continuing Care for Cocaine Dependence on HIV Sex-Risk Behaviors (2017) | Wimberly et al. | USA |
| The Effect of a Text Messaging Based HIV Prevention Program on Sexual Minority Male Youths: A National Evaluation of Information, Motivation and Behavioral Skills in a Randomized Controlled Trial of Guy2Guy (2018) | Ybarra et al. | USA |
| High Adherence to HIV Pre-exposure Prophylaxis and No HIV Seroconversions Despite High Levels of Risk Behaviour and STIs: The Australian Demonstration Study PrELUDE (2019) | Zablotska et al. | Australia |
| Effects of a Mobile Health Intervention to Promote HIV Self-testing with MSM in China: A Randomized Controlled Trial (2019) | Zhu et al. | China |
